# Cared for But Not Cared About? AI and the Moral Practice of Nursing

**DOI:** 10.1111/nup.70135

**Published:** 2026-10-26

**Authors:** Rachel Wangari Kimani

**Affiliations:** ^1^ Department of Law, School of Social Sciences The University of Manchester Manchester UK; ^2^ Decker College of Nursing and Health Sciences, Binghamton University Binghamton New York USA

**Keywords:** artificial intelligence, care ethics, engineered care, nursing ethics, patient experience, relational care

## Abstract

Artificial intelligence is increasingly deployed in healthcare not only to provide information or perform clinical tasks, but also to offer reassurance, companionship, and emotional support. This paper introduces the concept of engineered care to describe technological practices designed to generate the experiential effects of caregiving through system design rather than through the relational and moral practices that have traditionally constituted care. Its characteristic product is engineered comfort: the felt experience of reassurance, support, or being attended to that such systems can produce without assuming responsibility for the person receiving it. This comfort can be meaningful and may reduce distress, particularly where human care is scarce. The ethical challenge, therefore, is not simply that these systems fail, but that they succeed in producing experiences that resemble care. Drawing on care ethics, particularly Tronto's account of care as a moral practice, and on nursing's relational commitments, the paper argues that AI systems' growing capacity to produce comforting experiences risks redefining care as the delivery of desirable emotional outcomes. As reassurance and support become proxies for care, experiential indicators of success displace relational practices. Relational labor is increasingly delegated to technological systems, while responsibility for addressing the emotional and relational needs those systems leave unresolved continues to fall on nurses. Distinguishing engineered comfort from care enables nursing ethics to acknowledge these technologies' benefits without treating their experiential effects as equivalent to moral and relational practice.

## 1 Introduction

Patients increasingly receive reassurance, follow‐up, and emotional support from artificial systems. AI‐powered agents now conduct post‐discharge follow‐up, support chronic disease management, monitor symptoms, encourage medication adherence, and respond to patients seeking reassurance about their health(Bhimani et al. 2025; Hwang et al. 2025). These systems may not be intended to replace clinicians or assume professional roles. Rather, they are designed to produce many of the experiences patients associate with caring relationships: feeling heard, supported, reassured, and attended to. As such technologies become increasingly common, a new question emerges for nursing. If patients experience the effects of care, has care necessarily occurred? More fundamentally, can the experience of being cared for be separated from the caring practices that have traditionally made such experiences possible?

Nursing has long been understood as a relational profession, wherein emotional attentiveness, presence, and responsiveness are integral to its moral foundation rather than incidental aspects of care (Roy and Jones 2006). Professional frameworks codify this understanding: the International Council of Nurses Code of Ethics, on which many national codes are modeled, explicitly recognizes nurses' responsibility to provide compassionate, person‐centered care that addresses emotional and relational needs as well as clinical ones (International Council of Nurses 2021). However, nursing scholars have maintained that this relational base is rooted more in a moral and ontological duty than in professional obligation alone. Responsibility for the vulnerable other exists prior to any decision or agreement to provide care, positioning the nurse–patient interaction as a moral relationship first and a professional 1 s (Assem‐Erhaze 2026; Nguyen 2026; Watson 2005). This moral relationship is precisely what is challenged when technologies are engineered to replicate the experiential effects of care.

Much of the philosophical discussion of AI and care asks whether artificial systems can genuinely care. Scholars have argued that current systems cannot straightforwardly substitute for genuine caregiving because care requires moral agency, situated practical judgment, relational responsiveness, and answerability to the person receiving care (Ferdynus 2026; Wagner 2026). While these analyses are significant, they do not fully address the spectrum of ethical challenges encountered in healthcare practice. Numerous contemporary systems are not presented as moral agents or as complete replacements for human caregivers. Instead, they are primarily designed to generate experiences commonly associated with care, such as reassurance, companionship, and emotional support. The present inquiry concerns the implications when these effects become independently producible, scalable, and institutionally measurable.

These pressures are not new to nursing. In 2004, Jonsdottir et al. noted growing concern that nursing was becoming dominated by a technology‐driven, prescriptive, outcomes‐focused approach that overshadowed the caring relationship vital to helping people with complex health issues (Jonsdottir et al. 2004). What has changed is not the presence of technology in care, but the form that technological mediation now takes. Technologies have long helped nurses monitor patients, document care, coordinate services, and support clinical decision‐making. They altered how care was delivered without attempting to perform its relational work, a distinction that resonates with Locsin's argument that technology in nursing should support, not replace, the nurse–patient relationship (Locsin 2017).

However, a growing trend is toward technologies autonomously executing that relational work, and they are increasingly integrated into systems with every incentive to adopt them uncritically. The World Health Organization reports a global deficit of 5.8 million nurses World Health Organization (2025), and such shortages routinely justify adopting artificial agents to meet care needs in resource‐constrained settings. Beyond technology's influence on care, a distinct issue arises: can it still be regarded as care once stripped of the relational and moral work that has historically defined it?

This distinction becomes evident as care is increasingly evaluated based on the experience it provides. Patient‐experience metrics, for example, measure whether someone feels reassured, satisfied, or attended to, rather than capturing the practices that generate those feelings. Patients and families often define good and poor care based on whether comfort is provided or denied, making comfort a key indicator of healthcare quality and safety (Wensley et al. 2020). This attitude is not entirely new. Pérez warns that commercialization in healthcare encourages practitioners to view patients as clients seeking gratification rather than individuals who deserve proper care. This view is reinforced by care practices that quantify patient satisfaction, thereby reducing the reliance on relational judgment (Pérez 2014). Artificial companions and conversational systems are now assessed using the same criteria, focusing on emotional outcomes such as reassurance or loneliness reduction, which easily align with institutional metrics for demonstrating the effectiveness of healthcare technology (Broadbent et al. 2024; Loveys et al. 2022).

This is not an isolated tendency within healthcare; it mirrors a broader cultural pattern. Contemporary culture increasingly offers frictionless substitutes for emotionally demanding relationships. Beloved pets are cloned rather than grieved; in Kazuo Ishiguro's *Klara and the Sun* (2021), a mother contemplates having an artificial surrogate continue her dying daughter's identity rather than confront her loss (Ishiguro 2021). Romantic AI companions such as Replika now offer intimacy without vulnerability, connection without risk of rejection, loss, or demand (Pentina et al. 2023; Skjuve et al. 2021). What this paper calls ‘engineered care’ follows the same logic: it replicates the emotional effects of connection and comfort through design, making these feelings attractive by removing the challenges of human relationships. These include paying attention to another person whose reactions cannot be scripted, acknowledging them as an individual rather than a source of comfort, and embracing the mutual care that arises from this recognition.

In healthcare, this logic appears first not in dramatic interventions but in small, stabilizing gestures through which AI systems mimic care: inquiring about a patient's well‐being, drafting empathetic responses to messages in patient portals, suggesting reassuring language when patients describe symptoms, and reminding individuals to take medication and rest. These automated interactions reproduce many of the practices through which nurses convey relational attentiveness: recognizing distress, offering reassurance, and helping patients feel known and attended to rather than merely treated (DeFrino 2009). Because such responses appear supportive, produce measurable benefits, and are generally considered low risk, their ethical significance may be easily overlooked. Yet routine encounters are precisely where engineered responses can begin to substitute for relational work while remaining legible to patients and institutions as evidence that care has been provided.

Healthcare has long automated fragments of reassurance: the prewritten portal message, the standardized discharge instruction, the triage script. These are simple algorithms, and their comfort is thin and obviously canned. Artificial intelligence belongs to the same lineage but marks a step change within it. As an advanced form of algorithm, AI can generate responsive, personalized, emotionally attuned interactions that earlier automation could only gesture at: where a script delivers fixed words, an AI system can sustain something that feels like a relationship.

This paper introduces the concept of engineered care to name this development. The term builds on an earlier account of how AI reconfigures the moral labor of caregiving (Kimani 2026), extending it here to describe a substitutive form of care in which the goal is no longer cultivating caring relationships but producing care's emotional effects. Treating AI as the advanced case, this paper argues that as such systems succeed—and they often do—they risk redefining care as the production of comforting experiences, displacing the moral practices that have always distinguished caring from merely making someone feel cared for.

This distinction highlights three interconnected transformations in contemporary healthcare. First, care is increasingly assessed based on its experiential outcomes rather than its moral practices. Second, technology‐mediated systems redistribute the relational labor involved in caregiving. Third, the responsibility for repairing relational breakdowns is displaced onto nurses. Collectively, these shifts demonstrate that technological systems can subtly redefine the meaning of care, even when they ostensibly aim to enhance it. These transformations also organize the argument that follows. Section 2 situates the inquiry within nursing theory and scholarship on artificial intelligence in caregiving. Section 3 examines the first transformation: how engineered comfort shifts the assessment of care toward experiential outcomes. Section 4 addresses the second and third transformations, tracing how relational labor is redistributed within technology‐mediated systems and why relational repair continues to fall to nurses. Section 5 develops the implications for nursing ethics, education, and practice.

## 2 Engineered Care and the Conceptual Gap in Existing Scholarship

Nursing theorists have long understood care as an inherently relational and moral practice (Roy and Jones 2006; Swanson 1991; Watson 2005). This understanding is reinforced by broader care ethics scholarship, including the work of Tronto, Kittay, and Mol (Kittay 2019; Mol 2008; Tronto 1998). From this perspective, care is not something that happens to a patient but something that develops through presence, judgment, and responsibility for another's need. Therefore, when assessing a machine's usefulness in caregiving, it is important to look beyond just how well it performs tasks. Equally crucial is considering whether its use supports or diminishes the moral presence in the caregiving relationship.

Scholarship on artificial intelligence in caregiving environments has approached these questions from two broad directions. Design‐oriented frameworks focus on embedding ethical principles into the development of care technologies, for example, Sharkey and Sharkey's analysis of elder care robotics and van Wynsberghe's care‐centered value‐sensitive design (Sharkey and Sharkey 2012; Van Wynsberghe 2013). These approaches make important contributions to responsible innovation, but they tend to evaluate technologies by their capabilities and functions rather than by the moral significance of the comfort or reassurance they provide.

Relational analyses, by contrast, examine how people actually respond to artificial companions. Turkle's concept of relational artifacts and Coeckelbergh's argument that care can emerge within technologically mediated relationships explain why artificial systems can feel meaningful to those who interact with them (Coeckelbergh 2010; Turkle 2020). However, these analyses, while illuminating, stop short of asking what it means ethically when a patient experiences genuine comfort from an interaction that involves no moral accountability, no relational judgment, and no ongoing responsibility for their well‐being. Both traditions, in other words, describe what care technologies can do and how people respond to them, but neither fully examines what happens to care itself when its experiential effects can be reliably produced without its moral practices.

Within nursing scholarship, the relationship between technology and care has been debated directly. Locsin's theory of technological competency as caring holds that technology and caring are not opposed but complementary (Locsin 2017). When a nurse uses technology competently, it becomes an instrument through which the patient is known more fully. Nevertheless, this reconciliation depends on a nurse maintaining involvement in the relationship, i.e., technology demonstrates care because an accountable professional uses it. Engineered care names what happens when that condition fails: when technology no longer extends a nurse's attentiveness but stands in for it, producing the experience of being known without any professional remaining responsible for interpreting what is disclosed and acting on what it may signify. That nurses are nonetheless left holding what technology cannot supply is not new: Rubeis (2021) describes how nursing has long cast the nurse as a “guardian of humanity,” tasked with absorbing the human costs of technology that the system itself fails to address (Rubeis 2021). Reasoning from the same care‐ethics tradition, Paladino (2025) reaches the threshold of the present argument, observing that where reciprocity is absent, the care relationship becomes one‐directional and loses its meaning; yet she treats this as a risk to be managed by keeping the nurse in control of the tool (Paladino 2025). This paper advances the discussion by exploring what care entails when that control is, in practice, relinquished or when the experience of being cared for is created, and no nurse maintains a relationship at all.

Critical scholarship on AI‐mediated emotional support tends instead to characterize such interactions as illusory, deceptive, or morally insufficient (Kaczmarek 2025; Sparrow and Sparrow 2006). The most developed recent statement is Assing Hvidt et al.'s (2026) account of “empty empathy talk,” which shows that studies rating chatbot responses as more empathic than physicians' rely on unvalidated single‐item scales applied to decontextualized text, and concludes that automated empathy is a semantic shell, hollow for want of any embodied affective life (Assing Hvidt et al. 2026). The measurement critique is well taken. The diagnosis of hollowness, however, forecloses rather than opens the inquiry: dismissing engineered comfort as mere simulation leaves unexamined the moral consequences of an outcome that patients experience as real.

Zhan (2026) cultural case study offers a more productive starting point (Zhan 2026). Following a Chinese migrant woman navigating emotional distress across care infrastructures in China and the United Kingdom, Zhan conceptualizes AI‐mediated listening as a “care‐patch”: a provisional form of digital holding that becomes valuable where human listening is scarce, delayed, or experienced as burdensome. The user's sense of being heard is not dismissed as false simply because the system lacks embodied affect. Instead, it is situated within culturally specific relations of obligation, gendered expectations, and uneven access to human support. This account supports the claim that AI‐generated comfort may be experientially real while also directing attention beyond the interaction itself. If the chatbot functions as a patch, the issue becomes which systemic or institutional gaps this substitution is filling, what it permits institutions to leave unrepaired, and what long‐term consequences follow when provisional support becomes normalized as care.

To pursue those consequences, several distinctions are necessary. Agency refers here to the capacity to make a difference to what occurs. Conversational AI exercises functional agency insofar as it generates responses, shapes interpretation and action, and materially affects the encounter. Agency may also be distributed across a sociotechnical arrangement comprising the user, system, designers, deploying institution, and surrounding care services (De Togni et al. 2021). Moral agency makes a stronger claim: it entails the capacity to recognize moral reasons, form moral commitments, and undertake obligations toward another. Therefore, a system's causal influence on an encounter and its participation in a broader sociotechnical network do not automatically make it a moral agent. Responsibility here refers to the forward‐looking obligation, central to Tronto's account, to ensure that an identified need is adequately met. Accountability refers to the institutional requirement that identifiable actors explain and justify decisions, be evaluated against relevant standards, and answer for what was done or left undone. Agency may therefore be distributed, but responsibility must be differentiated and accountability assigned. As Hagendorff (2022) argues, distributing responsibility across technological and social networks does not mean responsibility vanishes.

This is most visible where comfort recurs without anyone assuming responsibility for assessing its wider significance. Consider a deliberately simplified case: a university student experiencing anxiety feels overwhelmed and turns at midnight to a mental health chatbot provided by their university. The system is immediately available, and disclosing distress to it may feel easier than approaching another person. The AI responds empathetically, and the student feels heard, which lessens their distress enough to help them sleep. The comfort is genuine. Emerging evidence suggests that university students can perceive chatbots as providing emotional support and that such interactions may reduce anxiety and particular forms of digital stress, although evidence of longer‐term benefit remains limited (Nyakhar and Wang 2025; Yang et al. 2026). However, the interaction does not establish that anyone has adequately assessed the severity or evolving meaning of the student's distress, assumed professional responsibility for their well‐being, or exercised relational judgment to determine what care they may need. Describing the interaction as merely deceptive would therefore overlook its actual effects and the relief the student experienced. Indeed, because such interactions can work experientially for users and operationally for institutions, they become plausible substitutes for human support.

Viewed through Tronto's account, the interaction contains partial analogs of several phases of caring (Tronto 2020). The system appears attentive because it recognizes and responds to the student's expressed distress. Its response may produce immediate relief, and the student's improved state provides evidence of care‐receiving and responsiveness. But these moments do not form an integrated caring practice. The system does not possess a moral interest in the student's welfare, exercise relational judgment about the meaning of their recurring distress, or assume responsibility for ensuring that their need is adequately met.

What, then, has occurred? The student‐‐AI encounter is neither merely deceptive nor straightforwardly caring. It exemplifies what this paper calls engineered care: the deliberate production of experiences associated with being cared for through a system that cannot itself assume the moral and relational responsibilities of caring. The design‐focused and relational frameworks considered earlier provide limited tools for examining this combination of experiential effectiveness and absent moral responsibility. This gap is especially consequential in nursing when nurses are expected to supply the judgment, continuity, and accountability that such systems lack, despite having limited authority over their design, procurement, or deployment.

This analysis builds on earlier work showing that integrating AI reconfigures the moral labor of caregiving across Tronto's phases of attentiveness, responsibility, competence, and responsiveness, redistributing its activities across designers, institutions, and technical systems (Kimani 2026). The present paper isolates one consequence of that reconfiguration: the production of comfort without a corresponding assumption of responsibility by the system producing it. AI does not become a substitute for care simply because it can generate comfort. In various contexts, individuals may resort to chatbots because of provider unavailability, delay, cultural incongruence, or because they are experienced as burdensome (Limpanopparat et al. 2024; Spytska 2025). These systems may also offer immediacy, privacy, and easier disclosure. Zhan (2026) concept of the “care‐patch” captures how these advantages can make AI‐mediated support genuinely valuable within gaps in human care, without resolving the conditions that created those gaps.

Ethical considerations emerge when organizations or governing bodies interpret a chatbot's partial effectiveness as evidence of their provision of adequate support, rather than as an indication of unmet demand. This may occur when procurement decisions reward satisfaction or engagement without assessing continuity of care, chatbot provision is treated as an adequate response to insufficient human support, or reassurance is offered without a reliable route to professional review and escalation. Such arrangements may benefit institutions by absorbing unmet demand while leaving clinicians, particularly nurses, to manage needs and consequences that later re‐emerge. This paper therefore does not argue that technologically mediated comfort is inherently deficient or that AI‐generated comfort is ethically suspect because it is artificially produced. The critical threshold is reached when supplementation becomes substitution: when producing comfort is allowed to stand in for the moral practice of care. At that point, engineered comfort conceals rather than remedies what is absent, while nurses inherit responsibility for the consequences without corresponding authority over the conditions that produced them.

## 3 The Comfort of Engineered Care

When technological systems are deliberately designed to evoke reassurance, companionship, or emotional support, they can reproduce many of the experiential effects associated with caregiving relationships. This paper calls the institutional and technological phenomenon engineered care: technological practices that generate care‐like experiences, reassurance, emotional stabilization, or the feeling of being attended to, through system design rather than through the moral and relational practices that have traditionally structured caregiving. Its characteristic product is engineered comfort: the felt experience of reassurance or affective stabilization that such systems produce in patients. The distinction is between practice and experience — engineered care names what systems and institutions do; engineered comfort names what patients feel as a result. Through conversational scripts, affect‐sensitive interfaces, and structured interaction patterns, individuals may feel calmer, heard, or less alone, even when interacting with systems that assume no responsibility for their well‐being.

Nursing ethics is precise about what caring practice requires. Tronto's influential framework highlights four essential moral practices: attentiveness, which involves continually recognizing others' needs; responsibility, meaning taking on the obligation to meet those needs; competence, the ability to respond effectively; and responsiveness, the capacity to stay accountable to the cared‐for individual as their circumstances change (Tronto 1998). In nursing, these are tangible professional commitments rather than abstract principles. Attentiveness involves understanding a specific patient, not just patients generally; responsibility requires identifiable persons or organizations to assume obligation for the patient's care; competence entails applying clinical and relational judgment to the current situation; and responsiveness requires attending to what follows. Engineered care can produce recognizable indicators of these elements, but the appearance of attentiveness or responsiveness does not, in itself, establish that the underlying obligations have been assumed.

Early mental health chatbots such as Woebot and Wysa rely largely on rule‐based architectures that guide users through structured therapeutic dialogue using predefined scripts or decision‐tree algorithms (Darcy et al. 2021; Malik et al. 2022). These systems simulate elements of therapeutic conversation by prompting users with reflective statements or follow‐up questions and offering cognitive‐behavioral therapy exercises, such as mood tracking or reframing negative thoughts. Phrases such as “That sounds really difficult” or “I'm glad you reached out today” can produce an experiential signature of attentiveness: the user feels noticed, responded to, and understood. That effect may be beneficial. The ethical distinction is that producing the experience does not itself identify who has interpreted the person's situation, assumed responsibility for what follows, or judged whether a different response is required.

While these dynamics are most observable in consumer mental health applications, they are increasingly evident within nursing practice itself, manifesting in the conversational scripts integrated into patient portal systems, the reassurance language incorporated into discharge instruction templates, and the AI‐powered triage tools that merge clinical assessment with standardized emotional support. Message drafting of this kind is one of the most extensively studied of these practices, and the comparisons in that literature, chatbot against clinician, rated for empathy on a five‐point scale, have consistently favored the generated text (Assing Hvidt et al. 2026). These ratings suggest the message reads as attentive, a property of the message rather than of anyone's attention to the patient. In each instance, the care‐delivery infrastructure has been engineered to provide comfort. It has not been designed to provide the moral accountability that genuine care requires. Without such accountability, no individual bears responsibility for assessing whether the comfort provided was appropriate, whether it alleviated the concerns of a patient who, in reality, required escalation, or whether it reassured someone whose deterioration went unnoticed. While the comfort itself may be genuine, the protective mechanism inherent in authentic care remains absent.

Clinical studies suggest that such systems may reduce symptoms of depression and anxiety in the short term and can foster forms of therapeutic alliance that users perceive as supportive (Jia et al. 2025; Lim et al. 2022). Reviews report modest therapeutic benefits and limited evidence of sustained effects, alongside user frustration with repetitive, generic, or constrained responses (Haque and Rubya 2023; He et al. 2023). Even when these systems work, a person may genuinely feel supported, heard, and less distressed without anyone taking responsibility for interpreting and responding to their needs.

As AI systems gain technical capacity, this distinction becomes more significant. Recent advances in generative AI facilitate more seamless, personalized, and emotionally responsive digital mental health platforms that are increasingly being integrated into earlier therapeutic systems. (Bean et al. 2026; Uscher‐Pines et al. 2026). These improvements may strengthen users' experiences of support without establishing who is obligated and equipped to act when further care is needed. For nursing ethics, this matters because increasingly convincing expressions of attentiveness may make it harder to distinguish the experience of being cared for from a practice in which someone assumes responsibility for one's care.

One reason engineered comfort can be persuasive lies in the reduced relational friction characteristic of human‐machine interaction. Unlike human caregivers, artificial systems do not contradict, tire, withdraw, or impose reciprocal emotional demands. Instead, they respond predictably, maintain consistent attentiveness, and avoid many of the ambiguities, misunderstandings, and negotiations that often accompany human relationships (Kirk et al. 2025). Patients may be drawn to these systems not despite knowing they are artificial, but in part because their artificiality removes the unpredictability, reciprocal demand, and emotional exposure that genuine caregiving relationships require. Engineered systems seem compassionate not because they engage in moral relationships of care, but because they replicate some of the experiential effects of care while eliminating its relational requirements. For nurses, this matters in a specific professional sense. The relational demands that engineered systems remove — the negotiation, the contradiction, the sitting with uncertainty — are not incidental features of caregiving that technology helpfully eliminates. They are the practices through which nurses exercise moral judgment, build therapeutic relationships, and fulfill their professional accountability to patients.

Of Tronto's four elements, responsibility warrants particular attention. In nursing, professional standards, scopes of practice, and patient assignments help establish who is responsible for responding to a patient's needs. Yet caring responsibility also extends beyond formally specified duties. Van Nistelrooij and Visse (2019) describe its receptive dimension: caregivers may find themselves called to respond to another person's vulnerability in ways they had not anticipated or been explicitly assigned. Their account helps explain why completing an assigned task does not necessarily settle what care requires. In an encounter with a conversational AI system operating without direct professional involvement, a person may disclose distress and receive effective reassurance while no one takes responsibility for understanding what that distress means or whether further support is needed. The question is who attends to that need and takes responsibility for responding when reassurance alone is insufficient.

Responsible AI scholars, such as Dignum (2019), emphasize that responsibility is rooted in the larger socio‐technical system. This requires attention to the people, practices, and institutions involved in AI's development and application. Dodig‐Crnkovic et al. (2025)propose a functional perspective on responsibility, enabling its allocation among human and artificial agents while retaining ultimate human responsibility for the system. Rashid (2026) examines how retrieval‐augmented generation (RAG) systems that can execute actions through external tools complicate responsibility attribution, arguing for explicit arrangements for layered answerability across people and institutions. These accounts illustrate how responsibility might be allocated, but their adequacy for care also depends on what counts as fulfilling it. A chatbot may successfully perform its assigned function by offering reassurance, while the institution records a satisfactory interaction without establishing whether the person's need has been adequately addressed. From a care‐ethical perspective, completing the designated task does not necessarily discharge responsibility for the need that prompted it.

This concern about who takes responsibility when reassurance is built into a system extends beyond conversational AI to routine healthcare delivery infrastructures. Triage decision trees, algorithmically guided care pathways, and standardized communication templates frequently incorporate reassurance alongside clinical guidance (Al Kuwaiti et al. 2023). For example, triage algorithms often combine risk assessment with scripted language intended to reduce patient anxiety while advising monitoring or follow‐up care (Zhao et al. 2026). Patient portals deliver laboratory results with prewritten messages designed to reassure patients when findings are normal or mildly abnormal. Discharge instructions generated through electronic health record systems include standardized statements regarding expected symptoms and typical recovery trajectories. In each case, reassurance is not provided by an individual but is embedded within the system. Moreover, when reassurance is embedded within the system, the erosion of responsibility inherent in genuine caregiving is embedded as well.

This broader shift can be understood in both institutional and interactional terms. Scholars of platform governance have shown that technological systems increasingly serve as foundational infrastructures, shaping interactions across multiple domains. Van Dijck describes this process as platformization, in which digital platforms function like a branching tree whose roots extend into diverse institutional practices, embedding standardized rules, metrics, and interaction patterns within everyday activities (Van Dijck 2021). When similar infrastructural logics enter healthcare, caring interactions may be organized around comparable principles. Emotional reassurance, attentiveness, and support can be specified in advance as system outcomes, standardized through interfaces and protocols, and delivered consistently across settings. Engineered care involves more than just individual tools; it requires a comprehensive reorganization of how care is provided, acknowledged, and assessed.

Evidence of benefit prompts another question: what do these outcomes indicate about the standards for care practices? In a randomized controlled trial involving 100 surgical patients, Yahagi et al. (2024) compared ChatGPT‐mediated nursing consultations with conventional nurse‐guided consultations during preoperative care. Patients in the AI‐supported group reported significantly lower anxiety scores, clearer information, and greater confidence in decision‐making. Rubin et al. (2024) likewise discuss how users may experience emotional understanding and support through interactions with AI‐driven therapeutic systems. Such benefits matter to those receiving support.

When these outcomes are treated as sufficient evidence that care has been provided, comfort can come to stand for the adequacy of care as a whole. Patient‐experience metrics, satisfaction surveys, and engagement indicators may reinforce this tendency if organizations use them to evaluate care without also examining its relational and moral dimensions. Positive experiences may then be recorded without establishing who is responsible for responding to the person's needs.

This separation between experienced support and caring responsibility also has a relational dimension. Ruggieri et al. (2026) depiction of an “uncanny setting” offers a way to interpret encounters in which familiar signs of a therapeutic relationship are present while the reciprocity, recognition, and responsibility associated with those signs remain indeterminate. In the student's encounter described above, empathetic language and immediate relief make the interaction recognizably care‐like, although the chatbot has undertaken no obligation toward the student. Engineered care names the production of this experience; the uncanny setting draws attention to how the disjunction between therapeutic appearance and moral structure may be experienced.

But why should this disjunction matter if the interaction reduces distress, satisfies the person using it, and sustains engagement? Drawing on Coeckelbergh's argument that human moral life depends on the appearance of emotion and moral presence rather than direct access to another's inner states, one might contend that a system that convincingly produces the appearance of care has thereby entered the moral domain (Coeckelbergh 2010). If the student feels heard, experiences less distress, and willingly returns to the system, what ethically significant good is missing?

This objection treats comfort as the primary good at stake and the caring relationship as merely one means of producing it. Cribb and Entwistle's account of “relationship goods” clarifies why care ethics resists this reduction: goods such as recognition, responsiveness, trust, mutuality, and being cared for are constituted within relationships and cannot be reduced to the psychological outcomes those relationships produce (Cribb and Entwistle 2026). To be cared for is therefore not simply to experience reassurance but to stand within a relationship in which another has recognized a need and assumed some responsibility for responding to it. The relationship is part of the good of care, not merely an instrument for producing a desired emotional state. This argument does not depend on detecting an inner state the system lacks; a demand that, as Gunkel notes, we could not reliably meet even for a human caregiver (Gunkel 2026). It depends instead on a more publicly ascertainable question: who, if anyone, has assumed responsibility for this patient's needs? A person comforted by a system without an identifiable actor responsible for recognizing and responding to what follows may experience real relief while remaining outside a relation of accountable concern. As these systems become more convincing, that absence may become harder to perceive. Floridi (2026) describes a form of semantic pareidolia in which, beyond a threshold of resemblance, people cease distinguishing simulated concern and begin attributing genuine care to the artifact.

This transformation is not the product of any single technology. Rather, it emerges through a broader structural reorganization of care. Engineered care can therefore be understood through three defining structural features: design pre‐specification, standardization, and scalability (see Figure 1). Design pre‐specification makes emotional reassurance an anticipated system outcome. Standardization embeds care‐like interactions in protocols and algorithms rather than negotiating them wholly through ongoing relationships. Scalability allows the experiential effects of care to be reproduced across users and settings. Together, these features show how technological systems can create experiences associated with care while making the relational practices and allocations of responsibility behind those experiences less visible. From a user's perspective, the resulting comfort may be difficult to distinguish from comfort received within a caring relationship; ethically, however, the surrounding structure of obligation remains relevant.

**Figure 1:**
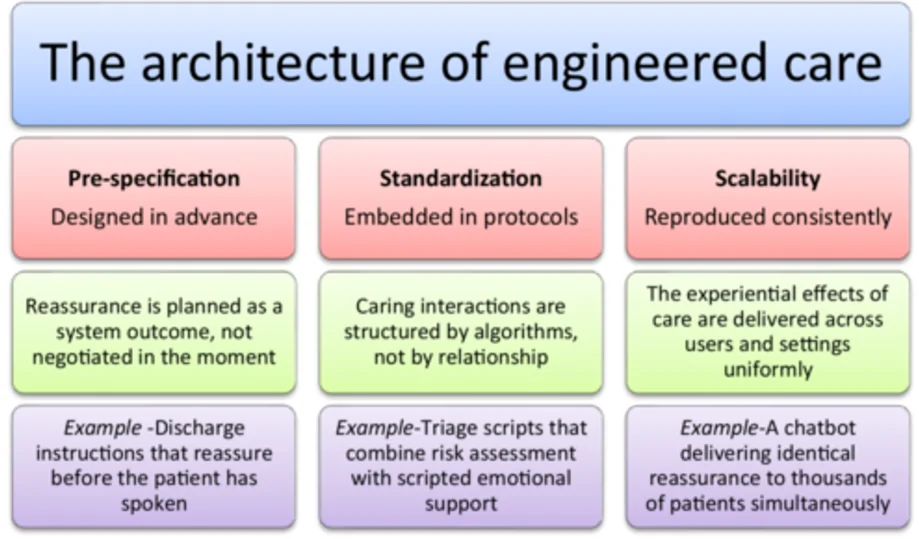
Engineered care is not a single tool or intervention but a structural phenomenon constituted by these three interlocking features, each of which transforms the conditions under which care is practiced and evaluated.

## 4 Engineered Care and the Redistribution of Relational Labor

If engineered care transforms how care is experienced, it also reshapes who performs the relational work that care requires. Alison Pugh's account of connective labor provides a useful lens for understanding this transformation (Pugh 2024). In The Last Human Job, Pugh describes how contemporary service systems increasingly rely on human workers to provide emotional presence, reassurance, and relational continuity in contexts where interactions have already been standardized, automated, or optimized. Rather than eliminating relational work, technological systems often intensify the demand for it by shifting the responsibility for repairing breakdowns in automated interactions onto human workers.

Within the context of engineered care, this redistribution is closely tied to the production of engineered comfort. Because engineered systems can successfully generate experiences of reassurance or attentiveness, they may create the impression that care has already occurred, even when the relational and moral work of caregiving remains unresolved. Engineered comfort can therefore obscure the ongoing human labor required to interpret distress, rebuild trust, and respond to needs that exceed the scope of standardized interactions.

This dynamic is particularly salient for nursing practice. When protocols, conversational scripts, decision‐support tools, or artificial companions provide reassurance, nurses may be left to restore meaning and relational continuity where that reassurance proves insufficient. Their work expands to include interpreting distress beyond standardized responses, explaining algorithmic recommendations, and addressing moral questions the interaction leaves unresolved. Drawing on the ethics of care, Stokes and Palmer (2020) propose managing this division by reserving the tasks most closely tied to caring for human nurses and recommending that a nurse remain present for the activities where touch and presence matter most.

An emerging example is using generative AI systems to conduct patient‐facing nursing calls at scale, including post‐discharge check‐ins that review medication instructions, probe for new or worsening symptoms, and answer patient questions (Gamble 2026; Hippocratic 2026). Such systems are marketed as a shift from scarcity to abundance. Their developers locate accountability in human oversight, where the nurse selects the patient, initiates the call, and reviews the result, while the system conducts the interaction. The vendor summarizes this arrangement as “the AI is the tool; the nurse is the clinician” (Hippocratic 2026).

This oversight assigns nurses a supervisory role, but its adequacy depends on the authority and resources that accompany it. Reviewing a completed call may allow a nurse to identify unresolved needs; acting on them requires time, access to relevant information, and workable routes for intervention and follow‐up. Responsibility must also be differentiated among nurses, deploying organizations, and developers, since each controls different aspects of the encounter. If nurses are held responsible for outcomes driven by design, deployment, and procurement choices beyond their control, formal oversight might hold them accountable without giving them the authority to fulfill that responsibility.

The practical limits of this oversight become clearer when the system also determines what information carries forward between encounters. The developers describe an agent that autonomously manages conversational memory, selecting which information from prior encounters to retain and later surface during subsequent calls (Sanz Ausin et al. 2026). These selections shape the context of later interactions and may influence which needs become visible to the reviewing nurse. Oversight therefore requires more than reviewing a completed call; it also requires sufficient visibility into how the system selects and uses the person's history. Alnawafleh et al. (2025) systematic review of AI in nursing practice identifies broader difficulties in attributing accountability, relevant to arrangements in which nurses are expected to answer for outcomes shaped by processes they have limited ability to inspect or influence. As advanced autonomous systems assume more control over the interaction, preserving a nurse's formal place in the workflow does not by itself ensure that the nurse can understand, question, and redirect how the person's needs are addressed.

This limited control may be accompanied by an uneven recognition of nurses' contributions. When engineered care produces comfort, its apparent success may obscure the human work that supports it: interpreting distress, clarifying misunderstandings, and sustaining trust. DeFrino (2009) describes how nurses' relational work can be institutionally “disappeared,” despite its importance to patients' experience of care. Applied to engineered care, this analysis suggests that benefits attributed to the system may depend partly on nursing work that remains unrecognized.

When the interaction breaks down, that same work may become an explicit expectation. Nurses may be called upon to address unmet expectations, unresolved distress, or misunderstandings generated by system‐mediated interactions. When they are also held answerable for failures beyond their control, this resembles what Elish (2019) describes as a moral crumple zone, in which responsibility for failures in automated systems falls disproportionately on nearby human actors. In engineered care, nurses' work remains unacknowledged when systems operate successfully; however, when systems fail, they become expected to fix issues, inheriting breakdowns from interactions they had little authority to influence.

A related displacement appears in Kropf (2026)'s “unlucky nurse” scenario, although the failure concerns medication administration rather than reassurance. A care robot administers fatal medication after a nurse documents a concern manually but fails to update its electronic record. The scenario highlights how accountability can shift to the nurse's obligation to keep the machine informed. For this paper's argument, it illustrates the need to examine how duties to technical systems reshape nurses' responsibilities to patients and whether the resulting allocation gives sufficient weight to system design and organizational conditions.

## 5 Ethical Implications for Nursing Practice

The ethical challenge posed by engineered care becomes evident when considering the nature of the nursing profession. Hugman (2014) distinguishes between established professions, which care about their clients through the skilled application of expert knowledge, and caring professions, which integrate caring about and caring for into the work itself. In nursing, moral commitment shapes how caregiving tasks are understood and performed. Offering reassurance involves judging whether it is warranted, attending to how it is received, and remaining answerable for what follows. When engineered systems' comforting functions are treated as equivalent to nursing care, nursing is then understood as though its tasks could be detached from the moral responsibilities enacted through them. Separating the comforting response from these obligations fundamentally alters the nature of the practice. What is eliminated constitutes an essential element of nursing care, even when the ensuing interaction maintains its comforting outcomes.

The implications of engineered care for nursing practice, however, are not straightforward. One might reasonably argue that the shifts described in this paper represent a legitimate and ethically defensible adaptation of caregiving practices rather than a problematic redefinition of care. Care has always been mediated by technologies, protocols, and institutional constraints, and outcomes such as reduced loneliness or emotional stabilization are themselves morally significant (Sandelowski 2000). If artificial systems reliably produce comfort in contexts of scarcity, distinguishing sharply between caring and care‐like outcomes might appear to privilege idealized relational models over practical well‐being. Moreover, moral responsibility in healthcare is already distributed across teams and institutions, suggesting that the absence of accountability within the technology itself does not necessarily negate the value of the support it provides.

This is not an argument against technology in care. Barnard and Sandelowski (2001)caution that the persistent polarization of technology and humane care may serve professional identity interests more than patient welfare, and that technology is often deliberately enrolled in the service of caring practice rather than opposed to it. The caution is well taken, and the distinction this paper draws is narrower than that of opposition. Technology enrolled in the service of care, such as a monitor that alerts a nurse to a deteriorating patient, a portal that lets a patient reach the team outside visiting hours, extends the reach of relational and moral practice. Engineered care reproduces the experiential effects of care while operating independently of the practices that give those effects their meaning. The concern is not mediation but substitution: not technology used as a temporary measure in conditions of scarcity, but comfort installed as the standing measure of whether care has occurred.

The first implication concerns where responsibility lies. Because the moral core of nursing cannot be detached from its relational practice, a system that produces a comforting response does not thereby assume responsibility for the patient, although it may create the appearance that the patient's need has been recognized and addressed. Responsibility for the moral adequacy of care therefore does not disappear when systems are designed to generate comfort; it becomes displaced and fragmented. Nurses may still be expected to interpret patients' needs, respond to resulting distress, and address concerns generated by systems they neither designed nor can meaningfully interrogate. Engineered comfort thus separates the production of a caring effect from the ongoing responsibility to attend and respond, while increasing the moral burden borne by nurses.

A second implication concerns the threshold for what counts as adequate care. When comfort, emotional stabilization, or satisfaction are treated as sufficient indicators that care has occurred, the relational and interpretive dimensions central to nursing practice are easily overshadowed. This does not make comfort ethically insignificant; it underscores that comfort alone cannot substitute for the attentiveness, responsibility, and responsiveness that constitute caring relationships. The danger lies in allowing care‐like outcomes to stand in for care itself.

A third implication concerns professional identity and moral distress. As nurses are positioned as ethical backstops for system‐designed interactions, they may experience tension between institutional expectations and their own understanding of good care (Corley 2002). Repairing relational gaps, clarifying misunderstandings created by artificial agents, and responding to unmet emotional needs can intensify moral distress, particularly when time, staffing, or organizational constraints prevent nurses from responding as fully as they believe patients require. Engineered care may therefore contribute to moral distress not by eliminating caring work, but by reshaping it under conditions that make ethically adequate care increasingly difficult to sustain.

A fourth implication concerns the possible erosion of relational competence. Caring capacities are not static personal attributes but practical abilities cultivated through repeated acts of attentiveness, interpretation, judgment, and response. When these dimensions of care are increasingly delegated to artificial systems, nurses may have fewer opportunities to develop and exercise the relational judgment through which good care is recognized and enacted. Similar concerns have been raised in medicine, where the increasing use of AI‐generated empathic communication has been argued to risk diminishing opportunities for clinicians to exercise and cultivate empathic practice (Assing Hvidt et al. 2026). Sustained reliance on engineered interactions may likewise narrow the relational practices through which nursing competence develops and is maintained. Engineered care may therefore contribute to a form of relational deskilling in which nurses remain responsible for the moral adequacy of care while having fewer opportunities to cultivate the capacities that responsibility requires.

These implications also matter for how artificial systems are introduced and taught in clinical practice. Emerging AI tools are frequently framed in educational and institutional contexts as supportive technologies that enhance comfort, efficiency, or patient satisfaction (Barnard and Sandelowski 2001). Clinicians and nurses are encouraged to view such systems as extensions of care rather than as ethical interventions requiring scrutiny. Yet education that presents these systems only as extensions of care may obscure their role in redistributing responsibility, redefining adequacy, increasing relational repair work, and narrowing opportunities for relational judgment. Nursing education must therefore address not only how these systems function, but also what forms of professional practice they may displace, what obligations remain with nurses, and how relational capacities can be preserved when parts of caring work are technologically mediated.

Engineered care matters for nursing because its adoption can reshape what counts as nursing work, which capacities are cultivated, and who is expected to answer for patients' needs. When comforting outcomes become the measure of successful care, the judgment, moral presence, and relational work that sustain nursing may receive less institutional recognition and support. Evaluating these technologies therefore requires attention to whether the arrangements surrounding their use preserve nurses' capacity and authority to respond to the person. Decisions about engineered care are also decisions about the conditions under which nursing can continue to be practiced as a caring profession.

## 6 Conclusion

This paper has argued that when technological systems produce comfort, reassurance, or a sense of being attended to, those effects are not in themselves sufficient to establish that care has occurred. The concept of engineered care names the gap between the experiential effects associated with caregiving and the moral and relational practices that constitute care, a gap that healthcare systems and professionals cannot afford to overlook.

Engineered care gains institutional traction because it aligns with institutional priorities of consistency, availability, scalability, standardization, and measurable outcomes. Technologies that produce reassurance or emotional stabilization fit readily within healthcare systems oriented toward efficiency. This compatibility is precisely what makes engineered care ethically significant. When comfort becomes a primary indicator of successful care, the relational and moral practices of caregiving can recede from view: affirmed in professional commitments and patient‐experience measures, yet insufficiently staffed, supported, or recognized in everyday practice.

This invisibility has a structural dimension. Engineered care does not eliminate moral responsibility; it disperses responsibility across institutional stakeholders while obscuring who remains accountable for the consequences. When systems appear to provide care, the relational labor that sustains that appearance is concealed, leaving nurses to interpret distress algorithms cannot detect, rebuild trust beyond scripted interactions, and assume accountability for moral gaps never built into the system. As engineered interactions become more seamless, this work may become more difficult to identify, even as its burden increasingly falls on nurses.

Recognizing engineered care as a distinct ethical phenomenon is important because it preserves the distinction between the comfort a system produces and the moral practice of care. As healthcare becomes increasingly technologically mediated, ethical inquiry should move beyond asking whether artificial systems can care and instead examine how care‐like effects are produced, which moral practices they displace or conceal, and who remains responsible for what follows. The practices of attentiveness, responsibility, competence, and responsiveness must remain visible, valued, and institutionally supported. Future inquiry should also consider whether relational care depends upon forms of reciprocity, vulnerability, or interpersonal demand that engineered systems are designed to minimize or cannot experience.

For nursing, the stakes are particular. Moral commitment to the patient is a core practice through which nursing is enacted. When engineered systems reproduce the experiential effects of care without assuming its obligations, nurses remain responsible for recognizing unmet needs, repairing relational gaps, and responding to concerns generated within systems in which they are not meaningful participants in design and governance. Unless the experience of care is distinguished from the practice of caring, institutions will read comfort as evidence that care has occurred, obscuring the relational and moral commitments that make nursing what it is. The patient may experience being cared for. Whether the patient is cared about remains a separate question. For nursing, preserving that distinction is essential because its professional identity rests not only on what is done for patients, but on the moral commitment through which that work becomes care.

## Author Contributions

The author conceptualized and wrote the manuscript.

## Funding

The author has nothing to report.

## Ethics Statement

Not applicable. This article is a conceptual analysis and does not involve human participants or empirical data.

## Consent for Publication

Not applicable. No direct human or human data was involved in the study.

## Conflicts of Interest

The author declares no conflicts of interest.

## Data Availability

Data sharing not applicable to this article as no datasets were generated or analysed during the current study.
